# SpoVG Is a Conserved RNA-Binding Protein That Regulates *Listeria monocytogenes* Lysozyme Resistance, Virulence, and Swarming Motility

**DOI:** 10.1128/mBio.00240-16

**Published:** 2016-04-05

**Authors:** Thomas P. Burke, Daniel A. Portnoy

**Affiliations:** a Department of Molecular and Cell Biology, University of California, Berkeley, Berkeley, California, USA; b School of Public Health, University of California, Berkeley, Berkeley, California, USA

## Abstract

In this study, we sought to characterize the targets of the abundant *Listeria monocytogenes* noncoding RNA Rli31, which is required for *L. monocytogenes* lysozyme resistance and pathogenesis. Whole-genome sequencing of lysozyme-resistant suppressor strains identified loss-of-expression mutations in the promoter of *spoVG*, and deletion of *spoVG* rescued lysozyme sensitivity and attenuation *in vivo* of the *rli31* mutant. SpoVG was demonstrated to be an RNA-binding protein that interacted with Rli31 *in vitro.* The relationship between Rli31 and SpoVG is multifaceted, as both the *spoVG*-encoded protein and the *spoVG* 5′-untranslated region interacted with Rli31. In addition, we observed that *spoVG*-deficient bacteria were nonmotile in soft agar and suppressor mutations that restored swarming motility were identified in the gene encoding a major RNase in Gram-positive bacteria, RNase J1. Collectively, these findings suggest that SpoVG is similar to global posttranscriptional regulators, a class of RNA-binding proteins that interact with noncoding RNA, regulate genes in concert with RNases, and control pleiotropic aspects of bacterial physiology.

## IMPORTANCE

*spoVG* is widely conserved among bacteria; however, the function of this gene has remained unclear since its initial characterization in 1977. Mutation of *spoVG* impacts various phenotypes in Gram-positive bacteria, including methicillin resistance, capsule formation, and enzyme secretion in *Staphylococcus aureus* and also asymmetric cell division, hemolysin production, and sporulation in *Bacillus subtilis*. Here, we demonstrate that *spoVG* mutant strains of *Listeria monocytogenes* are hyper-lysozyme resistant, hypervirulent, nonmotile, and misregulate genes controlling carbon metabolism. Furthermore, we demonstrate that SpoVG is an RNA-binding protein. These findings suggest that SpoVG has a role in *L. monocytogenes*, and perhaps in other bacteria, as a global gene regulator. Posttranscriptional gene regulators help bacteria adapt to various environments and coordinate differing aspects of bacterial physiology. SpoVG may help the organism coordinate environmental growth and virulence to survive as a facultative pathogen.

## INTRODUCTION

The Gram-positive bacterium *Listeria monocytogenes* is a facultative intracellular foodborne pathogen that can infect many organisms, including humans (1). *L. monocytogenes* occupies an unusually large ecological niche, thriving in environmental water sources, soil, decaying plant matter, and in other diverse habitats (2, 3). *L. monocytogenes* is also a well-adapted pathogen that grows rapidly in the cytosol of host cells. *L. monocytogenes* pathogenesis depends on the master transcriptional regulator PrfA, a Crp family member that regulates virulence gene expression (4). Pathogenesis also requires robust resistance to lysozyme, a potent antibacterial molecule of the innate immune system that is found throughout the body of all animals (5, 6).

Many bacterial pathogens, including *L. monocytogenes*, are highly lysozyme resistant due to a constitutive upregulation of cell wall enzymes that are conserved among both pathogens and nonpathogens, including PgdA (peptidoglycan deacetylase A), OatA (*O-*acetyltransferase A), and PbpX (penicillin-binding protein X) (7–9). We previously performed a forward genetic screen to identify lysozyme-sensitive *L. monocytogenes* mutants, and we found a highly abundant noncoding RNA, *rli31*, whose mutation led to significantly decreased lysozyme resistance (7). Small noncoding RNAs (sRNAs) are an emerging class of regulators in bacteria that primarily alter gene expression by imperfectly base-pairing at or near the ribosome-binding site (RBS) of target mRNA (10). A small number of sRNAs have also been shown to interact with proteins, often leading to inhibition of their function (11). Upon characterizing the *rli31* mutant phenotype, we determined that lysozyme sensitivity was due to decreased *pgdA* and *pbpX* mRNA abundance, and suppressor mutations that upregulated *pgdA* were sufficient to restore lysozyme resistance to a Δ*rli31* strain (7). However, Rli31 contained no detectable complementarity to *pgdA* or *pbpX* transcripts, suggesting that the relationship between these molecules is indirect.

Here, we again attempted to identify an Rli31 target(s) by identifying lysozyme resistance suppressor mutations via whole-genome sequencing. These suppressor strains were derived in the *pgdA* mutant background in order to circumvent the identification of mutations that upregulated *pgdA.* Upon genome sequencing, we observed that four of the five individually derived strains contained an identical mutation in the promoter of an operon encoding two copies of the gene *spoVG*, resulting in its significant downregulation. *spoVG* is broadly conserved, especially among Gram-positive bacteria (12), and *spoVG* mutants display remarkable phenotypes in many species, including reduced methicillin resistance, decreased capsule production, and decreased enzyme secretion in *Staphylococcus aureus* (13–15) and altered asymmetric cell division, decreased hemolysin production, and sporulation phenotypes in *Bacillus subtilis* (16–18). Additionally, our lab identified *spoVG* in a separate suppressor screen for mutants that rescued virulence defects of (p)ppGpp-deficient *L. monocytogenes* (19). Despite these phenotypes and despite being initially characterized nearly 40 years ago (20), the function of the *spoVG*-encoded protein has remained unclear. In this study, we determined that SpoVG is an RNA-binding protein that interacts with noncoding RNAs, regulates genes in cooperation with RNases, and controls pleiotropic aspects of bacterial physiology, including motility, carbon metabolism, and virulence. Together, these characteristics are similar to those of posttranscriptional gene regulators such as CsrA, a class of RNA-binding molecules that are fundamental for synchronizing environmental cues with gene regulation in order to adapt bacteria to their diverse ecological domains (11). Considering that these proteins have been primarily characterized in Gram-negative bacteria, we hypothesize that SpoVG may act as a functionally conserved counterpart to these molecules in Gram-positive organisms.

## RESULTS

### Suppressor analysis of lysozyme-sensitive mutants.

Previous attempts to select lysozyme-resistant suppressor mutants in the *rli31* mutant background identified mutations that upregulated *pgdA* (7). To identify other genes involved with lysozyme resistance, here we generated five independently derived lysozyme-resistant suppressor strains in the *pgdA* mutant background. Whole-genome sequencing and variant analysis identified differences between these suppressor strains and the parental *pgdA* strain (Table 1). All five strains encoded mutations in the essential *walRK* two-component system (TCS) operon, which upregulates expression of autolysins and other cell wall components (21, 22). Three mutations mapped to the response regulator *walR*, one mapped to the histidine kinase *walK*, and one mapped to *walI*, a negative regulator of *walRK* that we previously characterized as a lysozyme-sensitive mutant in *L. monocytogenes* (7). These data suggest that increased activation of the WalRK TCS leads to lysozyme sensitivity, while reduced activation leads to increased lysozyme resistance. It is unlikely that WalRK is a direct Rli31 target, however, as *walI* mutants display gross morphological cell wall phenotypes, such as susceptibility to antibacterial peptides and β-lactam antibiotics (7).

**TABLE 1:** Variants identified by genome sequencing of lysozyme-resistant Δ*pgdA* suppressor strains

Suppressor strain	Position on 10403S chromosome	Nucleotide		lmo number	Gene name	Mutation
		Reference	Alteration			
Δ*pgdA* #1	307762	G	T	*lmo0287*	*walR*	Gly92Tyr
Δ*pgdA* #2	194393	T		*lmo0196*	*spoVG*	14 nt 5′ of TSS
	312197	A	G	*lmo0290*	*walI*	Thr220Ala
	1788717	A	C	*lmo1759*	*pcrA*	Gly30Gly
Δ*pgdA* #3	194393	T		*lmo0196*	*spoVG*	14 nt 5′ of TSS
	309675	G	A	*lmo0288*	*walK*	Met430Iso
	2151278–2151294		Insertion	*lmo2113*		Insertion
Δ*pgdA* #4	194393	T		*lmo0196*	*spoVG*	14 nt 5′ of TSS
	307741	C	T	*lmo0287*	*walR*	Ser85Phe
Δ*pgdA* #5	194393	T		*lmo0196*	*spoVG*	14 nt 5′ of TSS
	307792	C	T	*lmo0287*	*walR*	Thr102Met

In addition to the *walRK* mutations, four of the five suppressor strains contained an identical mutation in the promoter of the *spoVG* operon, 14 nucleotides (nt) upstream of the transcriptional start site. This mutation led to a 27-fold decrease in mRNA abundance compared to wild-type (WT) bacteria, as determined by quantitative real-time PCR (qPCR) (data not shown). The *spoVG* operon encodes two paralogs (with 84% identity to one another) of the gene *spoVG* (*lmo0196* and *lmo0197*). The function of *spoVG* and its relationship to Rli31 were unclear, but we observed that the *spoVG* 5′-untranslated region (UTR) contained 14/14 nucleotides of perfect complementarity to Rli31. Therefore, we chose to focus on understanding the relationship between SpoVG and Rli31.

### Mutation of *spoVG* increases *L. monocytogenes* lysozyme resistance.

**FIG 1:**
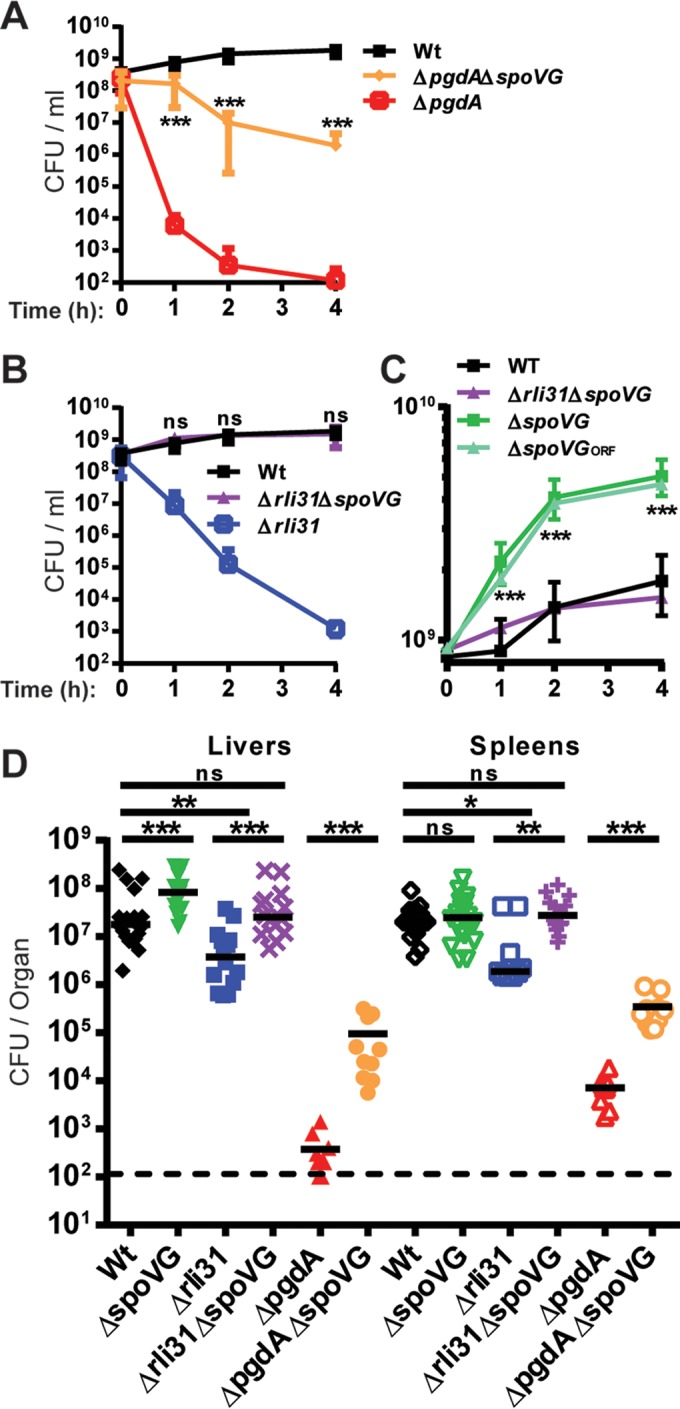
Deletion of *spoVG* increases lysozyme resistance and virulence of lysozyme-sensitive bacteria. (A, B, and C) The indicated strains were grown to mid-exponential phase with shaking in BHI and treated with 1 mg/ml lysozyme. CFU were measured at 1, 2, and 4 h post-addition of lysozyme. A two-tailed *P* value is reported for each *spoVG* mutant in comparison to its parental strain. ***, *P* < 0.0001; ns, no significant difference. (D) CFU in organs of CD-1 mice that were infected i.v. for 48 h. The data are a combination of the results of three separate experiments totaling at least 14 mice, with the exception of the Δ*pgdA* and Δ*pgdA* Δ*spoVG* strains, which were two separate experiments with a total of 10 mice each. The dotted line indicates the limit of detection, and black bars represent median values. A two-tailed Mann-Whitney *t* test was used for statistical analysis for each group. ns, no significant difference; *, *P* < 0.05; **, *P* < 0.01; ***, *P* < 0.0001.

A *spoVG* deletion mutant was constructed that lacked both *spoVG* paralogs and the 5′-UTR in the WT as well as the Δ*pgdA* and Δ*rli31* mutant backgrounds. These mutants were assayed for lysozyme sensitivity along with their parental strains. Deletion of *spoVG* significantly increased lysozyme resistance of the *pgdA* mutant (Fig. 1A) and completely restored lysozyme resistance for the Δ*rli31* strain (Fig. 1B). Deletion of *spoVG* in an otherwise-WT background increased lysozyme resistance to a level greater than that in WT bacteria (Fig. 1C). These data suggest that Rli31 and SpoVG each regulate lysozyme resistance in the absence of the other, but deletion of both genes leads to a neutral, WT phenotype.

Given the intriguing complementarity between Rli31 and the *spoVG* 5′-UTR, we examined whether the *spoVG* 5′-UTR affected lysozyme resistance. A chromosomal mutation in the *spoVG* 5′-UTR was introduced that disrupted the region’s complementary to Rli31. However, this mutation did not alter the degree of lysozyme resistance observed in the parental strain and did not affect other observable phenotypes (data not shown). In-frame deletion of the *spoVG* open reading frames (ORFs), which left the 5′-UTR intact (here referred to as the *spoVG_ORF_* mutant) (19), was similar to the *spoVG* mutant lacking the UTR (Fig. 1C). These findings suggest that the *spoVG* mutant phenotype is caused by mutation of *spoVG* and not by the *spoVG* 5′-UTR.

To investigate the role of each *spoVG* paralog, premature stop codons were introduced into *spoVG I* and *spoVG II*, and these strains were assayed for lysozyme resistance. Mutation of either paralog in the *rli31* mutant background did not significantly change lysozyme sensitivity of the *rli31* mutant (data not shown), suggesting that the function of the paralogs is redundant. This likely explains why suppressor mutations were identified in the promoter of the operon, which reduced expression of both paralogs, rather than a single *spoVG* ORF.

### Mutation of *spoVG* increases virulence *in vivo.*

To determine if *spoVG* contributed to growth *in vivo*, infections in mice were performed using Δ*spoVG*, Δ*spoVG* Δ*rli31*, and Δ*spoVG* Δ*pgdA* strains. Deletion of *spoVG* significantly increased *in vivo* growth of the Δ*pgdA* strain in both spleens (40-fold rescue) and livers (174-fold rescue). Deletion of *spoVG* also restored virulence of the *rli31* mutant (5-fold below WT) back to WT levels in both spleens and livers. In addition, a *spoVG* mutant in a WT background was 5-fold more virulent than WT bacteria (Fig. 1D). These data demonstrated that deletion of *spoVG* increases the growth *in vivo* of lysozyme-sensitive and WT *L. monocytogenes.*

### Characterization of the Rli31 secondary structure.

**FIG 2:**
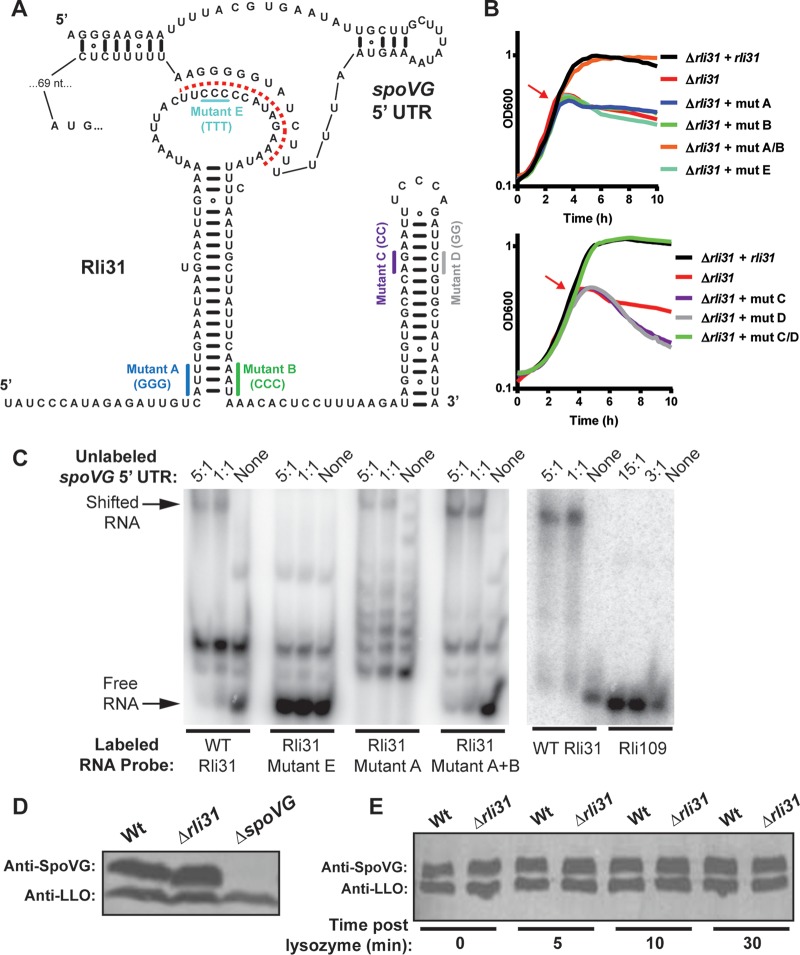
Rli31 binds the *spoVG* 5′-UTR *in vitro* but does not regulate *spoVG* expression. (A) The secondary structure of Rli31 and the *spoVG* 5′-UTR, as predicted by using RNAfold (51). The red dotted line indicates complementarity between the RNAs. (B) The mutations indicated in panel A were introduced into *rli31* on the pIMK plasmid and integrated into Δ*rli31* strain bacteria. Cultures were treated with 1 mg/ml lysozyme at mid-exponential phase, as indicated by the red arrow. Turbidity was monitored at 10-min intervals. Data are representative of results from at least three separate experiments. (C) Approximately 400 ng of the indicated ^32^P-labeled *in vitro*-transcribed RNA was incubated with unlabeled *spoVG* 5′-UTR RNA at 25°C for 30 min. EMSAs were performed using native PAGE. Molar ratios of unlabeled 5′-UTR to ^32^P-labeled RNA are indicated. (D and E) *L. monocytogenes* lysates were collected from the indicated strains, separated by gel electrophoresis, and imaged by Western blotting using an antibody specific for SpoVG (17). A nonspecific band that reacted with an antibody to LLO was used as a loading control. (E) Lysozyme (200 μg/ml) was added to mid-logarithmically growing bacteria in BHI cultured with shaking at 37°C. Bacteria were then harvested at the indicated times, and Western blot assays were performed as described for panel D.

The *spoVG* 5′-UTR contained a higher degree of complementarity to Rli31 (14/14 nucleotides) than anywhere else on the *L. monocytogenes* chromosome (Fig. 2A). To better understand this relationship, we characterized the predicted Rli31 secondary structure. Expression of WT *rli31* with its endogenous promoter fully complemented lysozyme sensitivity of the Δ*rli31* mutant (Fig. 2B). Mutations that disrupted the 5′ hairpin (mutant A or B) or the 3′ hairpin (mutants C or D) were then introduced into *rli31*, which failed to complement the Δ*rli31* mutant (Fig. 2B and C). Mutations in the 5′ apical loop (mutant E) also failed to complement the Δ*rli31* strain. Compensatory mutations that restored the 5′ hairpin (mutant A+B) or the 3′ transcriptional terminator (mutant C+D) restored lysozyme resistance of Δ*rli31* to WT levels (Fig. 2B). These results support the predicted structure of Rli31 and suggest that Rli31 is composed of a long 5′ hairpin and a 3′ transcriptional terminator. The complementary region between Rli31 and the *spoVG* 5′-UTR is encoded in a C-rich apical loop of Rli31.

### Rli31 binds the *spoVG* 5′-UTR *in vitro* but does not regulate SpoVG mRNA or protein abundance.

Given the complementarity between Rli31 and the *spoVG* 5′-UTR, we asked whether these RNAs interacted *in vitro*. RNA-RNA electrophoretic mobility shift assays (EMSAs) were performed using ^32^P-labeled *in vitro*-transcribed (IVT) WT and mutant Rli31 and the unlabeled *spoVG* 5′-UTR. Addition of the *spoVG* 5′-UTR caused a migration difference of WT Rli31 at a molar ratio of 1:1 but did not alter migration of Rli31 mutant E at a molar excess of 5:1 (Fig. 2C). The *spoVG* 5′-UTR also altered migration of Rli31 mutant A+B, while Rli31 mutant A showed an altered migration pattern compared to WT Rli31 (Fig. 2C). Another RNA of similar size (Rli109) was also tested as a negative control, and addition of the *spoVG* 5′-UTR to Rli109 did not cause a migration difference when we used a molar excess of 15:1 (Fig. 2C, right panel). These data suggest that the *spoVG* 5′-UTR and the Rli31 apical loop specifically interact *in vitro.*

We hypothesized that Rli31 regulates *spoVG* transcript or protein abundance. To evaluate whether Rli31 affects *spoVG* transcript stability, qPCR was performed using RNA from WT and Δ*rli31* bacteria. However, mRNA abundance of *spoVG* was unaltered in the *rli31* mutant, and *rli31* RNA abundance was unaltered in *spoVG* mutants (data not shown). To test if *rli31* affected *spoVG* protein abundance, Western blot assays were performed using WT and *rli31* mutant bacteria. Surprisingly, no difference in SpoVG protein abundance was observed between WT and *rli31* mutants (Fig. 2D). Genes associated with lysozyme resistance are often upregulated in response to lysozyme (23, 24), so we also tested whether Rli31 regulated *spoVG* in response to lysozyme. A time course of lysozyme treatment revealed that SpoVG abundance was similar between WT and Δ*rli31* strain bacteria at all time points (Fig. 2E). These data suggest that, despite their sequence complementarity and despite interacting *in vitro*, Rli31 does not regulate *spoVG* mRNA or protein abundance.

### SpoVG weakly and nonspecifically interacts with single-stranded DNA *in vitro.*

**FIG 3:**
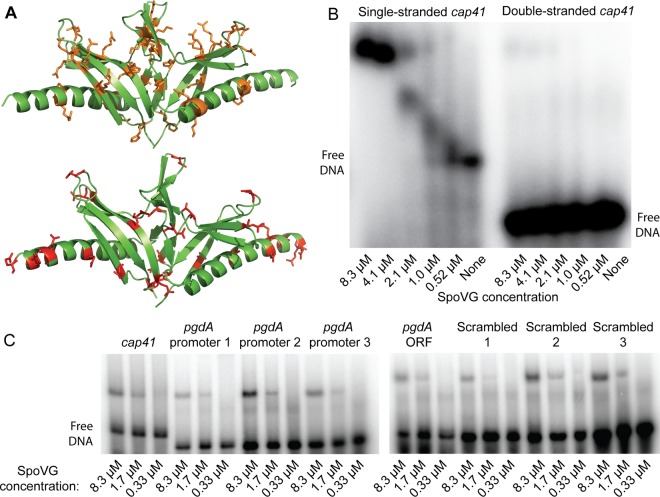
SpoVG binds weakly and nonspecifically to DNA. (A) The crystal structure of dimeric SpoVG from *B. subtilis* (PDB ID 2IA9). Peptide chains are annotated in green. Positively charged side chains are annotated and shown in orange in the top image, and negatively charged side chains are annotated and shown in red in the bottom image. (B and C) The indicated concentrations of SpoVG–6-His were incubated with 250 ng of the indicated ^32^P-labeled oligonucleotides for 30 min, as described in Materials and Methods, and separated by native PAGE. For double-stranded DNA, complementary *cap41* oligonucleotides were heated to 95°C and slowly cooled to room temperature for 1 h. (Oligonucleotide sequences are described in Table S3 in the supplemental material.)

*L. monocytogenes* SpoVG was previously reported to bind to DNA (25), and the crystal structure of *B. subtilis* SpoVG contained two positively charged grooves of similar widths (measured in Angstroms using PyMOL) (Fig. 3A). To assess DNA binding, SpoVG (Lmo0196) carrying a C-terminal six-histidine epitope tag was purified from *Escherichia coli*, and EMSAs were performed using ^32^P-labeled DNA*.* EMSAs were then performed with SpoVG and the *cap41* promoter from *S. aureus* (25) and the *pgdA* promoter from *L. monocytogenes* (oligonucleotide sequences are described in Table S3 in the supplemental material). The migrations of protein-DNA complexes were plotted against total protein concentrations, and nonlinear regression was used to calculate the apparent *K_d_* (dissociation constant) value for single-stranded *cap41* binding, which was 1.2 µM. Significant binding was not observed for double-stranded DNA (Fig. 3B). *spoVG* bound to various *pgdA* promoter probes with similar affinities as *cap41* and also bound to a probe corresponding to the *pgdA* ORF (Fig. 3C). These results suggested that DNA binding was not specific, and we observed that *spoVG* bound to various scrambled DNA probes with similar affinities as the *cap41* and *pgdA* probes (Fig. 3C).

### SpoVG binds multiple RNAs *in vitro*, including Rli31.

**FIG 4:**
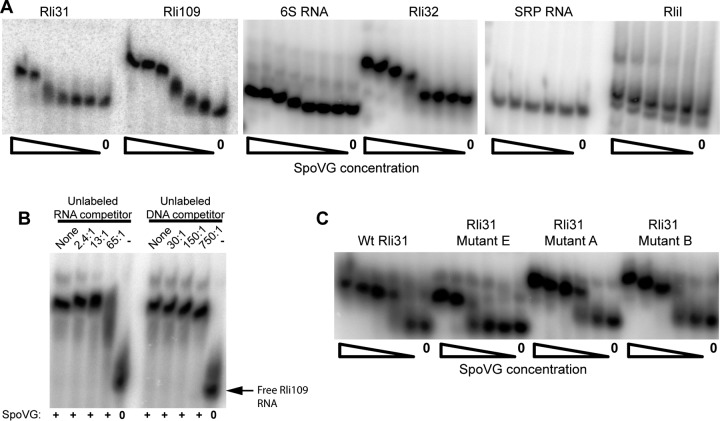
SpoVG interacts with RNA *in vitro*. (A, B, and C) EMSAs were performed using approximately 250 ng of the indicated ^32^P-labeled, *in vitro*-transcribed RNA and with the indicated concentrations of SpoVG–6-His. All EMSA reaction mixtures contained noncompetitive RNA/DNA/protein competitors (see Materials and Methods). (A) SpoVG–6-His concentrations for Rli31 and Rli109 were 1,030, 517, 259, 129, 65, 32, and 0 nM. SpoVG–6-His concentrations for 6S RNA and Rli32 reactions were 4,140, 2,070, 1,034, 517, 258, 129, 64.6, and 0 nM. SpoVG–6-His concentrations for SRP RNA and Rli109 were 2,070, 1,034, 517, 258, 129, and 0 nM. (B) Sixty nanograms of Rli109 was incubated with 258 nM SpoVG–6-His for 30 min with the indicated molar ratio of competitor RNA (Rli109) or DNA (*cap41*). The fifth and 10th lanes contained no SpoVG–6-His and no nucleotide competitor. (C) EMSA reactions were performed with 250 ng of WT Rli31 and the indicated Rli31 mutants, where the SpoVG–6-His concentrations were 2,070, 1,034, 517, 258, 129, and 0 nM.

Given the genetic interactions between *spoVG* and *rli31* and that SpoVG weakly bound DNA, we next asked if SpoVG interacted directly with Rli31. To assess RNA binding, *rli31* was *in vitro* transcribed and EMSAs were performed using SpoVG–6-His purified from *E. coli*. To ensure that SpoVG–6-His was not contaminated with Hfq, an RNA-binding protein that adheres to immobilized metal affinity columns (26), a two-step purification was performed that removed all detectable amounts of Hfq, as observed by Western blotting (see Fig. S1 in the supplemental material). EMSAs revealed that SpoVG bound Rli31 with an apparent *K_d_* of 273 nM. To test for specificity, five other *L. monocytogenes* noncoding RNAs were evaluated. RNAs were chosen that had well-established 5′ and 3′ ends and were of similar size to Rli31 (27, 28). These probes were 6S, Rli32, signal recognition particle (SRP) RNA, RliI, and Rli109. Two of these molecules, 6S RNA and SRP RNA, are well-described protein-binding RNAs, whereby the 6S RNA specifically interacts with proteinaceous RNA polymerase components (29) and the SRP RNA is part of the highly conserved signal recognition particle (30). Rli32, RliI, and Rli109 are uncharacterized sRNAs in *L. monocytogenes* (28). These RNAs were *in vitro* transcribed, EMSAs were performed, and gel shift assays showed a range of affinities for the various sRNAs (Fig. 4A). SpoVG bound to Rli109 with an apparent *K_d_* of 129 nM and to Rli32 with a *K_d_* of 273 nM. Very weak binding was observed for RliI, and no gel shifts were observed using the RNAs with known protein-binding partners, 6S RNA and SRP RNA (Fig. 4A).

These data suggested that SpoVG bound to RNA with a nearly 10-fold greater affinity than DNA. To directly compare RNA and DNA binding, competition assays were performed using radiolabeled RNA and unlabeled RNA/DNA competitors. SpoVG binding to ^32^P-labeled RNA (Rli109) was competed away using a 65:1 molar excess of unlabeled RNA (Rli109). However, a 750:1 molar excess of unlabeled DNA competitor (*cap41*) did not affect RNA binding of SpoVG (Fig. 4B).

To assess which region of Rli31 was bound by SpoVG, the Rli31 mutants described in Fig. 2 were *in vitro* transcribed and assayed for binding. Mutation of the Rli31 hairpin (mutants A and B) did not affect binding, while mutation of the apical loop (mutant E) resulted in 2- to 4-fold reduced binding (Fig. 4C). Collectively, these results revealed that *L. monocytogenes* SpoVG is an RNA-binding protein that interacts with the Rli31 5′ apical loop *in vitro*.

### *spoVG* mutant strains of *L. monocytogenes* are pleiotropic.

**FIG 5:**
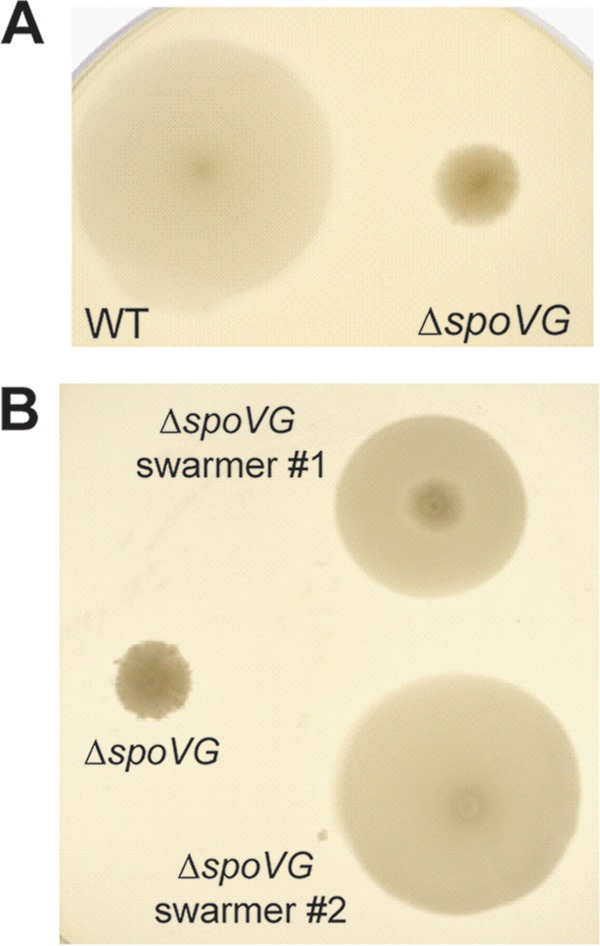
*spoVG* mutants are nonmotile in soft agar. (A) Aliquots (1 μl) of stationary-phase WT and Δ*spoVG* strain cultures were stab inoculated into 0.35% BHI agar plates. Bacteria were then grown for 72 h at 30°C. (B) Δ*spoVG* swarming suppressor mutant strains were isolated after prolonged growth at 30°C. Suppressor strains and the parental Δ*spoVG* mutant were then assayed for motility as described for panel A.

*spoVG* mutant strains of *S. aureus* are defective for secretion of extracellular enzymes (14). To characterize secreted proteins from Δ*spoVG L. monocytogenes* strains, supernatants from exponentially growing WT and Δ*spoVG* mutant bacteria were precipitated, separated by electrophoresis, and visualized by Coomassie staining. One band appeared for the WT that was lost in the Δ*spoVG* mutant, and mass spectrometry identified this protein as flagellin (see Fig. S2A in the supplemental material). Deletion of *flaA* in *L. monocytogenes* did not alter lysozyme resistance (data not shown); however, these data suggested that *spoVG* may be required for proper expression of motility genes. Δ*spoVG* appeared motile in liquid culture as observed by phase-contrast microscopy, but during growth in soft agar the *spoVG* mutant displayed a severe defect in swarming motility, spreading to only 6.6% of the area of WT bacteria (Fig. 5A). After prolonged incubation at 30°C, suppressors were observed that swarmed away from the original colony. These swarming suppressor mutants were isolated and reinoculated into soft agar. After 3 days, the suppressors displayed the smooth colony morphology and increased the swarming area to an average of 60% of WT (Fig. 5B). DNA from six individually derived swarming suppressors was purified and subjected to whole-genome sequencing. Four of the six strains contained unique point mutations in the gene encoding a major RNase, RNase J1 (Table 2). Two other mutations occurred in genes encoding RNA turnover machinery proteins, including the transcription termination protein Rho and the termination factor NusG (Table 2). The swarming suppressor mutations did not affect lysozyme resistance (see Fig. S2B), suggesting that *spoVG* independently regulates motility and lysozyme resistance.

**TABLE 2:** Variants identified by genome sequencing of Δ*spoVG* swarming suppressor strains

Suppressor strain	Position on 10403S chromosome	Nucleotide		lmo number	Gene name	Mutation
		Reference	Alteration			
Δ*spoVG* #1	2581931	G	T	*lmo2551*	*Rho*	Arg90Ser
	693327–693328		T	(Intergenic)	(Between *mogR* and *fliP*)	T insertion
Δ*spoVG* #2	258699	G	T	*lmo0246*	*nusG*	Val132Phe
	1037773	G	A	*lmo1027*	*rnjA* (RNase J1)	His364Tyr
Δ*spoVG* #3	2625645–2625689			*lmo2588*	*mdrT*	Rearrangement
	1038807	T	A	*lmo1027*	*rnjA* (RNase J1)	Glu19Val
Δ*spoVG* #4	581380–581479			*lmo0562*		Inversion
	<1914963–1915062			*lmo1885*		Inversion
	693327–693328		T	(Intergenic)	(Between *mogR* and *fliP*)	Insertion
Δ*spoVG* #5	1038270	T	G	*lmo1027*	*rnjA* (RNase J1)	Asn198Thr
	2622782	C	T	*lmo2586*		Val120Met
Δ*spoVG* #6	1037692	C	A	*lmo1027*	*rnjA* (RNase J1)	Gly391Cys

### SpoVG regulates carbohydrate import genes.

We sought to determine what genes were regulated by SpoVG in *L. monocytogenes*. Transcriptome sequencing (RNA-seq) was performed using RNA from Δ*spoVG* that was collected from mid-exponential-phase cultures growing at 37°C in brain heart infusion (BHI) medium. Compared to transcripts from WT bacteria, 577 genes were regulated greater than 2-fold and 101 genes were regulated greater than 4-fold in the *spoVG* mutant (see Table S1 in the supplemental material). Five of the 16 most upregulated genes encoded phosphotransferase system (PTS) components predicted to import carbohydrates (31). Of these, four genes were predicted to import *N*,*N*′-diacetylchitobiose, the disaccharide breakdown product of chitin. In summary, *spoVG* is required for proper expression of hundreds of *L. monocytogenes* genes, many of which are involved with carbohydrate metabolism.

## DISCUSSION

In this study, *spoVG* mutations were identified as suppressors of *L. monocytogenes* lysozyme sensitivity. *spoVG* is broadly conserved among bacteria (12) and has been extensively characterized for nearly 4 decades in Gram-positive bacteria (13, 14, 16, 17, 20, 32). *spoVG* mutations cause diverse phenotypes in *B. subtilis* and *S. aureus* related to capsule formation (15), sporulation (16, 17), enzyme secretion (14), antibiotic resistance (13), and cell division (17). In *L. monocytogenes*, *spoVG* mutants were also pleiotropic and were nonmotile, hyper-lysozyme resistant, hypervirulent, and contained upregulated carbon metabolism genes. Despite SpoVG often being regarded as a “regulator” (13, 15, 17, 33, 34), the mechanism by which it regulates gene expression has remained unclear. Here, we have shown that SpoVG binds RNA, and we suggest that the protein is a global posttranscriptional gene regulator in Gram-positive organisms.

Global posttranscriptional regulators, including the CsrA/Rsm family, are a class of RNA-binding proteins that coordinate various aspects of bacterial physiology and are primarily described in Gram-negative bacteria (11, 35). In this study, we observed a number of similarities between SpoVG and this class of regulators. For example, both *spoVG* and *csrA* mutants are pleiotropic and exhibit defects in motility, carbohydrate metabolism, and virulence (11). CsrA interacts with the noncoding RNA CsrB, which antagonizes its function (36). Similarly, SpoVG bound the apical loop of Rli31 *in vitro*. While it remains unclear how SpoVG regulates RNA transcripts, CsrA binds mRNA near the RBS to occlude ribosome recruitment. The binding affinity for SpoVG for Rli31 *in vitro* was weaker than that of CsrA (37); however, we note that Rli31 was among the most abundant RNAs in *L. monocytogenes* (7) and that the affinity of SpoVG for mRNA transcripts may be higher than for the arbitrarily chosen RNAs described in this report. Future studies will be required to determine the consensus binding motif and affinity of SpoVG for mRNA transcripts.

Another similarity between SpoVG and CsrA is the coregulation of genes with major RNases. CsrA regulates RNA transcripts by protecting targets from cleavage by RNase E, a major RNase in Gram-negative bacteria. CsrA can also remodel mRNA to unveil Rho-binding sites, leading to premature transcriptional termination (11). RNase E is not conserved in *Firmicutes*, including *L. monocytogenes*; however, the major RNases J1 and J2 are functional homologs (38). Interestingly, we found that point mutations in RNase J1, Rho, and NusG suppressed the swarming defects of *spoVG* mutants. We also noted that SpoVG is structurally similar to CsrA, as both proteins are small (less than 100 amino acids), dimeric, and consist of 5 to 7 β-strands and a single α-helix (11). Lastly, the *L. monocytogenes* spoVG operon encoded two highly similar *spoVG* paralogs, and each paralog was redundant for regulating lysozyme resistance and swarming motility. Likewise, multiple paralogs of CsrA are encoded in *Legionella pneumophila* and they regulate the organism’s ability to survive in unique habitats (39). Based on all these findings, we suggest that SpoVG may represent a global posttranscriptional regulator in Gram-positive bacteria, similar to the Csr/Rsm family of proteins described in Gram-negative organisms.

The Rli31 apical loop contained 14/14 nucleotides of perfect complementarity to the *spoVG* UTR. Not surprisingly, Rli31 interacted with the SpoVG 5′-UTR *in vitro*. Additionally, *spoVG* is regulated by noncoding RNAs in *S. aureus* (33, 40); therefore, we hypothesized that Rli31 regulates SpoVG translation. However, we were surprised to observe that Rli31 did not regulate SpoVG mRNA or protein abundance (Fig. 2D and E) and that mutating the *spoVG* 5′-UTR at the region of complementarity to Rli31 did not affect lysozyme resistance or swarming motility (data not shown). The function of the *spoVG* 5′-UTR remains unresolved; however, we speculate that the UTR negatively regulates Rli31 as an “RNA-RNA” decoy under certain growth conditions. In *Salmonella* and *E. coli*, RNA-RNA decoys sequester sRNAs through base-pairing, which regulates chitobiose import (41). Curiously, we observed that 4 of the 16 most upregulated genes in the Δ*spoVG* mutant were chitobiose import proteins. Based on the similarities between Rli31/SpoVG and the chitobiose decoy system described in proteobacteria, we speculate that the UTR may function as a means to regulate Rli31 in relation to carbohydrate import. Clearly, the relationship between Rli31 and SpoVG is exceedingly complicated and multifaceted, which may allow *L. monocytogenes* to fine-tune growth in complex environments.

*L. monocytogenes* lives as both a saprophyte and a foodborne pathogen. Its pathogenic lifestyle is controlled by the master virulence regulator PrfA, which is a Crp family member that is activated in host cells. PrfA mutants that are locked in the active state (PrfA*) are hypervirulent but suffer during environmental growth (42, 43). Since SpoVG regulates motility and carbohydrate metabolism, we suggest that it modulates *L. monocytogenes* physiology suitable for environmental survival. Additionally, we observed that *spoVG* mutants bind to the biofilm indicator dye Congo red significantly less than WT bacteria (data not shown), suggesting that SpoVG may positively regulate biofilm formation genes. Like PrfA* mutants, *spoVG* mutants were hypervirulent, and we suspect that, also like PrfA* mutants, they would suffer during environmental growth. The *spoVG* paralogs (*lmo0196* and *lmo0197*) occupy the locus adjacent to the PrfA regulon (*lmo0200* to *lmo0207*), which although possibly coincidental may suggest coregulation. Indeed, both genes are modestly regulated by SigB (44) and by PTS-dependent sugar abundance (45). We suggest that, whereas PrfA controls intracellular survival and pathogenesis, SpoVG tilts the balance toward environmental survival.

## MATERIALS AND METHODS

### Ethics statement.

This study was carried out in strict accordance with the recommendations in the *Guide for the Care and Use of Laboratory Animals* of the National Research Council of the National Academy of Sciences (46). All protocols were reviewed and approved by the Animal Care and Use Committee at the University of California, Berkeley (MAUP R235-0815B).

### Bacterial strains and microbiological assays.

All strains of *L. monocytogenes* used in this study were in the 10403S background and cultured in BHI medium. For construction of Δ*spoVG* mutant strain, a splice overlap extension product was generated with primers TB211/TB214 and cloned into pKSV7 (47), and *spoVG* was removed by allelic exchange. *rli31* complement strains were constructed by amplifying *rli31* and the *rli31* promoter with TB140 and TB141. This fragment was introduced into *L. monocytogenes* by using pIMK (48), and mutations were introduced using primers TB14-TB23. Transductions were performed using U153 phage as previously described (7, 49). Motility assays were performed as previously described (50). Hen egg white lysozyme (Sigma) was used for all lysozyme assays, and the assays were performed as previously described (7). Lysozyme-resistant *pgdA* suppressors were derived as described elsewhere (7) by passaging the Δ*pgdA* strain with increasing concentrations of lysozyme, until all strains were resistant to 1 mg/ml lysozyme. Lists of all strains and oligonucleotides used in this study are provided in Tables S2 and S3, respectively, in the supplemental material.

### *In vivo* infections.

All *in vivo* infections were performed with Crl:CD1(ICR) (CD-1) mice from Charles River. Mice were infected intravenously (i.v.) with 10^5^ logarithmically growing bacteria, and organs were harvested at 48 h postinfection. Organs were homogenized with 0.1% NP-40, and the indicated dilutions were plated onto LB agar.

### qPCR.

RNA was purified from 20 ml of logarithmically growing bacteria by phenol-chloroform extraction and ethanol precipitation. A 4.4-µg aliquot of RNA was DNase treated and reverse transcribed with iScript (Bio-Rad). cDNA levels were measured using SYBR Fast (KAPA) and oligonucleotides specific for the target gene (see Table S3 in the supplemental material).

### Whole-genome sequencing and RNA-seq.

Whole-genome sequencing and RNA-seq were performed as previously described (7) at the QB3 Functional Genomics Laboratory at UC Berkeley (http://qb3.berkeley.edu/qb3/fgl/). Sequence data were aligned using the CLC Genomics Workbench (CLC bio).

### Western blot analysis.

10 ml cultures of the indicated strains were grown to mid-exponential phase with shaking at 37°C in BHI medium, collected by centrifugation, and lysed by bead beating followed by boiling in SDS loading buffer. Protein abundance was normalized to the optical density at 600 nm (OD_600_), and soluble proteins were separated by denaturing gel electrophoresis. Membranes were probed using anti-SpoVG antibodies (Linc Sonenshein, Tufts University) and anti-listeriolysin O (LLO) antibodies. Membranes were then probed using the appropriate secondary antibodies (LI-COR), and fluorescence was visualized using an Odyssey imaging system (LI-COR).

### Modeling of SpoVG.

The crystal structure of SpoVG from *B. subtilis* (PDB accession number 2IA9) was manipulated using the PyMol molecular graphics system, version 1.7.4 (Schrödinger, LLC) to annotate positively charged residues (R/K/H) and negatively charged residues (E/D).

### Purification of SpoVG from *E. coli.*

*spoVG I* was amplified from the *L. monocytogenes* chromosome by using primers TB254/TB255, digested, ligated into pET20b, and transformed into BL21 cells containing pLysS. Bacterial cultures (1.4-liter cultures) were grown with shaking at 37°C until the OD_600_ reached 0.40, and *spoVG* expression was induced with 1 mM isopropyl-β-d-thiogalactopyranoside (Sigma) for 2 h. Bacteria were then collected by centrifugation, flash-frozen, and lysed by sonication in buffer A [300 mM NaCl, 50 mM Tris, 25 mM imidazole, 0.5 mM Tris(2-carboxyethyl)phosphine, 20% glycerol; pH 8.0]. Cell wall debris was removed by centrifugation, and the resulting lysate was passed over Ni-nitrilotriacetic acid (NTA) affinity resin (Thermo). The resin was washed with a minimum of 40 ml buffer A followed by elution with increasing concentrations of imidazole (50, 75, 100, 125, and 300 mM). Elutions 3 to 5 were pooled and dialyzed overnight into buffer B (1 mM dithiothreitol [DTT], 50 mM Tris-HCl, 25 mM KCl, 2 mM MgCl_2_, 10% glycerol, 1 mM phenylmethylsulfonyl fluoride; pH 8.0). Proteins were then separated by size exclusion chromatography using a Superdex 75 column (GE). Low-molecular-weight fractions (fractions 17 to 23) were pooled, concentrated using spin concentrators (Millipore), and evaluated in EMSAs. Protein concentrations were determined via the Bradford assay (Bio-Rad).

### EMSAs and nucleotide probe preparation.

For IVT reactions, PCR products containing T7 promoters were generated (see Table S3 in the supplemental material), and IVT was performed using [α-^32^P]ATP (PerkinElmer) and the MEGAscript T7 transcription kit (Life Technologies). IVT-derived RNAs were DNase treated, diluted in Tris-EDTA (TE buffer), and purified using MicroSpin G-25 columns (GE Life Sciences). EMSA reactions were performed with the indicated amounts of SpoVG in 25 to 30 µl volumes, and all reaction mixtures contained nonspecific DNA, RNA, and protein competitors. Unless otherwise stated, between 250 and 500 ng of ^32^P-labeled DNA/RNA was included for each reaction mixture. All reaction mixtures contained 2× EMSA buffer (40 mM Tris-HCl, 2 mM MgCl_2_, 20% glycerol, 300 mM NaCl, 5 mM DTT; pH 8.0), 5 µg yeast tRNA, 1 µg bovine serum albumin, and 100 ng poly(dI-dC). Reaction mixtures were incubated at room temperature for 30 min before separation by 5% native PAGE.

## SUPPLEMENTAL MATERIAL

Figure S1  Purification of SpoVG–6-His expressed by *E. coli*. (A) SpoVG with a 6-His epitope tag was purified by immobilized metal affinity chromatography (IMAC). The flowthrough, wash, and elutions were separated by SDS-PAGE and stained with nonspecific Coomassie brilliant blue. Elutions contained 50, 75, 100, 125, and 300 mM imidazole. The black bar represents fractions that were pooled, dialyzed, and analyzed by size exclusion chromatography (SEC). Lane 1 contains the ladder. (B) The elution profiles of pooled fractions from panel A on a Superdex 75 column. (C) Fifteen microliters of the indicated fractions from panel B were separated by SDS-PAGE and stained with Coomassie brilliant blue. Black bars indicate fractions that were pooled for Western blot analysis. (D) Five microliters of each SEC fraction was analyzed by Western blotting using an Hfq-specific antibody. Pooled fractions 10 to 16 and 17 to 23 were concentrated using spin concentrators prior to separation by SDS-PAGE. Download Figure S1, JPG file, 0.7 MB

Figure S2  *L. monocytogenes spoVG* mutants are defective for *flaA* secretion, which is independent from the Δ*spoVG* lysozyme resistance phenotype. (A) WT and Δ*spoVG* bacteria were grown shaking to mid-exponential phase in BHI at 37°C, bacteria were pelleted by centrifugation, and 2 ml of supernatant was precipitated with TCA. Supernatants were then separated with SDS-PAGE and stained with Coomassie Brilliant Blue. L indicates the ladder and the black arrow indicates the band which was excised, trypsin digested, and analyzed by mass spectrometry (UC Berkeley QB3). (B) The *pgdA*::Tn mutation was transduced into Δ*spoVG* and into the Δ*spoVG* swarming suppressor strains. Disk diffusions were performed with 1 mg lysozyme/disk. Means and standard deviations from at least 3 separate experiments are presented, where “***” indicates *P* < 0.001 and "(ns)" signifies no significant difference between the swarming strains and Δ*spoVG*, *pgdA*::Tn. Download Figure S2, JPG file, 0.68 MB

Table S1  Genes regulated in the Δ*spoVG* mutant strain with at least a 4-fold change from WT. (RNA-seq was performed with two technical replicates of WT and Δ*spoVG* bacteria. RNA was harvested and purified as described in Materials and Methods. Library construction and sequencing were performed at the Functional Genomics Laboratory at UC Berkeley. Data analysis was performed using CLC Genomics Workbench.) Table S1, DOCX file, 0.02 MB

Table S2  *L. monocytogenes* and *E. coli* strains used in this study (constructed as described in Materials and Methods). Table S2, DOCX file, 0.02 MB

Table S3  Oligonucleotides used in this study. Table S3, DOCX file, 0.01 MB
