# Supplementary figures and images for: SpoVG Is a Conserved RNA-Binding Protein That Regulates Listeria monocytogenes Lysozyme Resistance, Virulence, and Swarming Motility
Source: mBio. 2016 Apr 5;7(2):e00240-16. doi: 10.1128/mBio.00240-16 (PMC4959528; doi:10.1128/mBio.00240-16)

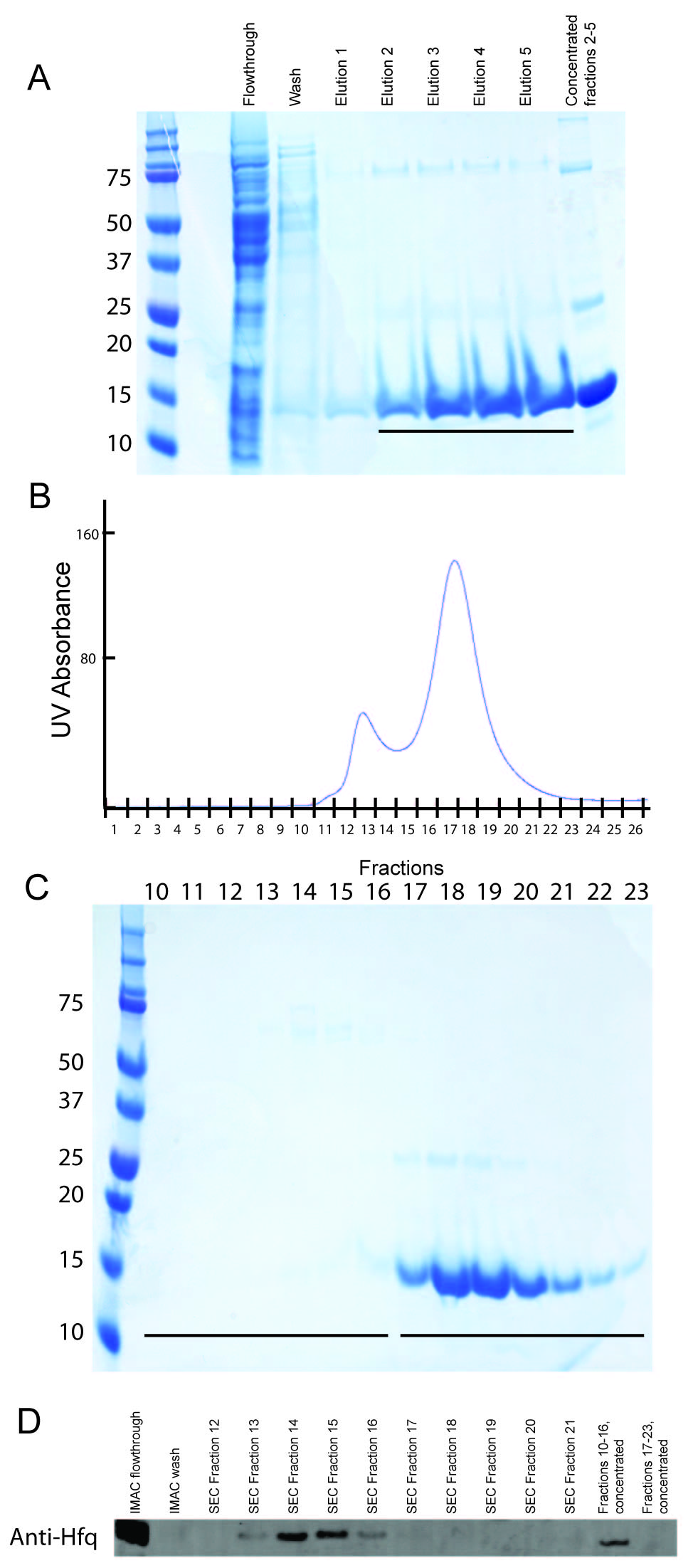

Supplement: Figure S1 — Purification of SpoVG–6-His expressed by E. coli. (A) SpoVG with a 6-His epitope tag was purified by immobilized metal affinity chromatography (IMAC). The flowthrough, wash, and elutions were separated by SDS-PAGE and stained with nonspecific Coomassie brilliant blue. Elutions contained 50, 75, 100, 125, and 300 mM imidazole. The black bar represents fractions that were pooled, dialyzed, and analyzed by size exclusion chromatography (SEC). Lane 1 contains the ladder. (B) The elution profiles of pooled fractions from panel A on a Superdex 75 column. (C) Fifteen microliters of the indicated fractions from panel B were separated by SDS-PAGE and stained with Coomassie brilliant blue. Black bars indicate fractions that were pooled for Western blot analysis. (D) Five microliters of each SEC fraction was analyzed by Western blotting using an Hfq-specific antibody. Pooled fractions 10 to 16 and 17 to 23 were concentrated using spin concentrators prior to separation by SDS-PAGE. Download [file mbo002162736sf1.jpg]

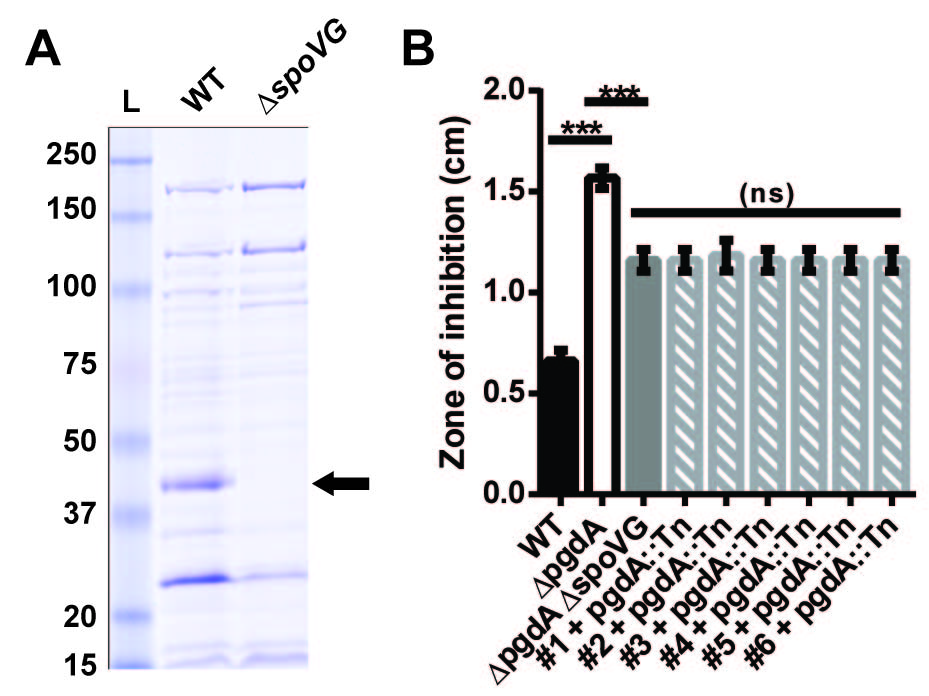

Supplement: Figure S2 — L. monocytogenes spoVG mutants are defective for flaA secretion, which is independent from the ΔspoVG lysozyme resistance phenotype. (A) WT and ΔspoVG bacteria were grown shaking to mid-exponential phase in BHI at 37°C, bacteria were pelleted by centrifugation, and 2 ml of supernatant was precipitated with TCA. Supernatants were then separated with SDS-PAGE and stained with Coomassie Brilliant Blue. L indicates the ladder and the black arrow indicates the band which was excised, trypsin digested, and analyzed by mass spectrometry (UC Berkeley QB3). (B) The pgdA::Tn mutation was transduced into ΔspoVG and into the ΔspoVG swarming suppressor strains. Disk diffusions were performed with 1 mg lysozyme/disk. Means and standard deviations from at least 3 separate experiments are presented, where “***” indicates P < 0.001 and "(ns)" signifies no significant difference between the swarming strains and ΔspoVG, pgdA::Tn. Download [file mbo002162736sf2.jpg]
