# Supplementary material for: SpoVG Is a Conserved RNA-Binding Protein That Regulates Listeria monocytogenes Lysozyme Resistance, Virulence, and Swarming Motility
Source: mBio. 2016 Apr 5;7(2):e00240-16. doi: 10.1128/mBio.00240-16 (PMC4959528; doi:10.1128/mBio.00240-16)
Supplement: Table S1 — Genes regulated in the ΔspoVG mutant strain with at least a 4-fold change from WT. (RNA-seq was performed with two technical replicates of WT and ΔspoVG bacteria. RNA was harvested and purified as described in Materials and Methods. Library construction and sequencing were performed at the Functional Genomics Laboratory at UC Berkeley. Data analysis was performed using CLC Genomics Workbench.) [file mbo002162736st1.docx]

| **Supplemental Table 1: Genes regulated in Δ*spoVG* at least 4-fold change from Wt** | | | | |
| --- | --- | --- | --- | --- |
| **Gene ID** | **10403s gene** | **Description** | | **Fold upregulation** |
| LMRG_01305 | *lmo1662* | Methyltransferase | | 18.7 |
| LMRG_00186 | *lmo0505* | Ribulose phosphate 3 epimerase, in Galactitol PTS operon | | 8.9 |
| LMRG_01487 | *lmo2356* | No known domains | | 8.8 |
| LMRG_00334 | *lmo0647* | No known domains | | 8.1 |
| LMRG_02569 | *lmo0299* | Similar to PTS beta-glucoside, N,N'-diacetylchitobiose enzyme IIB component. | | 7.8 |
| LMRG_01950 | *lmo2746* | No known domains | | 7.3 |
| LMRG_02014 | *lmo0914* | PTS system IIB component. Lactose, N,N'-diacetylchitobiose family | | 7.3 |
| LMRG_01622 | *lmo2210* | No known domains | | 7.3 |
| LMRG_01281 | *lmo2127* | CAAX protease | | 7.2 |
| LMRG_05064 | *lmo2824* | Similar to D-3-phosphoglycerate dehydrogenase | | 7.2 |
| LMRG_01917 | *lmo2780* | Similar to cellobiose PTS enzyme IIA. Lactose, N,N'-diacetylchitobiose family | | 6.9 |
| LMRG_02505 | *lmo1766* | PurN, similar to phosphoribosylglycinamide formyltransferases | | 6.9 |
| LMRG_01259 | *lmo2105* | Similar to ferrous iron transport protein B | | 6.9 |
| LMRG_01345 | *lmo1621* | Weakly similar to E. coli MutT protein | | 6.6 |
| LMRG_02388 | *lmo0139* | No known domains | | 6.2 |
| LMRG_02298 | *lmo0874* | PTS system enzyme IIA component. Lactose, N,N'-diacetylchitobiose family | | 6.2 |
| LMRG_01327 | *lmo1639* | Similar to dna-3-methyladenine glycosidase | | 6.1 |
| LMRG_05516 | *lmo2824* | Similar to D-3-phosphoglycerate dehydrogenase | | 6.1 |
| LMRG_01780 | *lmo2468* | ATP-dependent Clp protease proteolytic subunit | | 6.0 |
| LMRG_01343 | *lmo1623* | PAP2 superfamily | | 6.0 |
| LMRG_00927 | *lmo1474* | Heat shock protein GrpE | | 6.0 |
| LMRG_01943 | *lmo2753* | No known domains | | 5.9 |
| LMRG_00213 | *lmo0531* | GGDEF domain containing protein | | 5.8 |
| LMRG_00395 | *lmo0706* | flagellar hook-associated protein | | 5.5 |
| LMRG_02508 | *lmo1763* | Trp repressor protein | | 5.4 |
| LMRG_02387 | *lmo0138* | Domain of unknown function | | 5.4 |
| LMRG_00546 | *lmo1084* | Similar to DTDP-L-rhamnose synthetase | | 5.4 |
| LMRG_02259 | *lmo0836* | Phosphate-starvation-inducible protein PsiE | | 5.2 |
| LMRG_02128 | *lmo1028* | Similar to B. subtilis YkzG protein | | 5.2 |
| LMRG_02102 | *lmo1002* | PTS phosphocarrier protein Hpr (histidine containing protein) | | 5.1 |
| LMRG_02509 | *lmo1762* | No known domains | | 5.1 |
| LMRG_02094 | *lmo0994* | Domain of unknown function | | 5.0 |
| LMRG_01724 | *lmo2524* | Similar to hydroxymyristoyl-(acyl carrier protein) dehydratase | | 4.9 |
| LMRG_01017 | *lmo1870* | Similar to alkaline phosphatase | | 4.8 |
| LMRG_05063 | *lmo2824* | similar to D-3-phosphoglycerate dehydrogenase | | 4.8 |
| LMRG_00215 | *lmo0533* | ACT domain containing protein | | 4.8 |
| LMRG_02643 | *lmo0221* | Type III pantothenate kinase | | 4.8 |
| LMRG_00773 | *lmo1323* | Similar to B. subtilis YlxR protein | | 4.7 |
| LMRG_01263 | *lmo2109* | Alpha beta hydrolase fold containing protein | | 4.7 |
| LMRG_02196 | *lmo2651* | Similar to mannitol-specific PTS enzyme IIA component | | 4.6 |
| LMRG_00812 | *lmo1362* | Similar to exodeoxyribonuclease small subunit | | 4.6 |
| LMRG_01302 | *lmo1665* | No known domains | | 4.5 |
| LMRG_01363 | *lmo1604* | Similar to 2-cys peroxiredoxin | | 4.5 |
| LMRG_00316 | *lmo0633* | Similar to PTS system, fructose-specific IIB component. Fructose mannitol family. | | 4.5 |
| LMRG_02023 | *lmo0924* | Similar to ABC transporter, ATP-binding protein (C-terminal part) | | 4.4 |
| LMRG_00199 | *lmo0518* | Domain of unknown function | | 4.4 |
| LMRG_02934 | *lmo2222* | Calcineurin-like phosphoesterase superfamily domain | | 4.4 |
| LMRG_00518 | *lmo1056* | No known domains | | 4.4 |
| LMRG_02733 | *lmo2388* | Similar to B. subtilis YwqG protein | | 4.3 |
| LMRG_00271 | *lmo0589* | Domain of unknown function | | 4.2 |
| LMRG_01323 | *lmo1643* | No known domains | | 4.2 |
| LMRG_01893 | *lmo2803* | No known domains | | 4.2 |
| LMRG_00381 | *lmo0693* | Flagellar motor switch protein | | 4.2 |
| LMRG_00118 | *lmo0426* | Similar to PTS fructose-specific enzyme IIA component | | 4.2 |
| LMRG_01123 | *lmo1976* | Similar to oxidoreductase | | 4.1 |
| LMRG_01165 | *lmo2016* | CspB, cold shock protein | | 4.1 |
| LMRG_01988 | *lmo2709* | No known domains, small protein | | 4.1 |
| LMRG_02946 | *lmo1574* | dnaE | | 4.0 |
| LMRG_00489 | *lmo0800* | Similar to B. subtilis YqkB protein | | 4.0 |
| LMRG_00633 | *lmo1187* | Similar to ethanolamine utilization protein EutQ | | 4.0 |
| **Genes downregulated in Δ*spoVG,* at least 4-fold below Wt** | | | | |
| **Gene ID** | **10403s gene** | **Description** | **Fold downregulation** | |
| LMRG_02618 | *lmo0196* | spoVG I | -249.5 | |
| LMRG_01377 | *lmo1590* | argJ, bifunctional protein | -21.1 | |
| LMRG_01649 | *lmo2183* | Similar to ferrichrome ABC transporter (permease) | -15.9 | |
| LMRG_00712 | *lmo1263* | Similar to transcriptional regulator | -15.1 | |
| LMRG_02619 | *lmo0197* | spoVG II | -12.5 | |
| LMRG_01543 | *lmo2289* | Protein gp14 [Bacteriophage A118] | -9.0 | |
| LMRG_01647 | *lmo2185* | 3 NEAT domain containing protein, Iron transport protein | -8.8 | |
| LMRG_02805 | *lmo2135* | Similar to PTS system, fructose-specific enzyme IIC component. Fructose manitol family | -8.8 | |
| LMRG_01287 | *lmo1680* | Similar to cystathionine gamma-synthase | -8.6 | |
| LMRG_01261 | *lmo2107* | Hypothetical protein | -7.8 | |
| LMRG_00608 | *lmo1165* | Hypothetical protein | -7.8 | |
| LMRG_01672 | *lmo2160* | Xylose isomerase-like TIM barrel domain, endonuclease domain | -7.4 | |
| LMRG_01651 | *lmo2181* | Sortase B, SrtB | -7.3 | |
| LMRG_01117 | *lmo1970* | Similar to putative phosphotriesterase related protein | -7.3 | |
| LMRG_02806 | *lmo2134* | Similar to fructose-1,6-biphosphate aldolase type II | -7.2 | |
| LMRG_00019 | *lmo0326* | Similar to transcriptional regulators | -6.8 | |
| LMRG_01669 | *lmo2163* | Similar to oxidoreductase | -6.0 | |
| LMRG_01379 | *lmo1588* | ArgD, highly similar to N-acetylornithine aminotransferase | -6.0 | |
| LMRG_00592 | *lmo1149* | Similar to alpha-ribazole-5'-phosphatase | -6.0 | |
| LMRG_01300 | *lmo1667* | Similar to L-lactate dehydrogenases | -5.8 | |
| LMRG_00201 | *lmo0520* | Similar to transcription regulator | -5.8 | |
| LMRG_01652 | *lmo2180* | Siphovirus Gp157 | -5.7 | |
| LMRG_01650 | *lmo2182* | Similar to ferrichrome ABC transporter (ATP-binding protein) | -5.4 | |
| LMRG_01376 | *lmo1591* | argC | -5.3 | |
| LMRG_02626 | *lmo0204* | actA | -5.2 | |
| LMRG_00390 | *lmo0701* | No known domains | -5.2 | |
| LMRG_01726 | *lmo2522* | yocH | -5.1 | |
| LMRG_02678 | *lmo2589* | Similar to transcription regulator TetR/AcrR family | -5.1 | |
| LMRG_01872 | *lmo2826* | Similar to efflux proteins | -4.9 | |
| LMRG_00425 | *lmo0737* | No known domains | -4.7 | |
| LMRG_00594 | *lmo1151* | Similar to Salmonella typhimurium PduA protein | -4.7 | |
| LMRG_02627 | *lmo0205* | plcB | -4.7 | |
| LMRG_01524 | *lmo2321* | Protein gp45 of Bacteriophage A118 | -4.5 | |
| LMRG_02623 | *lmo0201* | plcA | -4.5 | |
| LMRG_00384 | *lmo0695* | Flagellar hook-length control protein FliK | -4.4 | |
| LMRG_00881 | *lmo1429* | Thiamine transporter protein (Thia_YuaJ) | -4.4 | |
| LMRG_01673 | *lmo2159* | Similar to oxidoreductase | -4.3 | |
| LMRG_00389 | *lmo0700* | Similar to flagellar motor switch protein fliY | -4.2 | |
| LMRG_02494 | *lmo0061* | No known domains | -4.1 | |
| LMRG_02492 | *lmo0061* | No known domains | -4.0 | |
| LMRG_02846 | *lmo0902* | Similar to transcription regulator (GntR family) | -4.0 | |
