# Supplementary material for: SpoVG Is a Conserved RNA-Binding Protein That Regulates Listeria monocytogenes Lysozyme Resistance, Virulence, and Swarming Motility
Source: mBio. 2016 Apr 5;7(2):e00240-16. doi: 10.1128/mBio.00240-16 (PMC4959528; doi:10.1128/mBio.00240-16)
Supplement: Table S2 — L. monocytogenes and E. coli strains used in this study (constructed as described in Materials and Methods). [file mbo002162736st2.docx]

| **Supplemental Table 2: Strains used in this study** | | | |
| --- | --- | --- | --- |
| **Strain description** | **Reference** | | **Strain #** |
| WT 10403S | | (1) |  |
| Δ*rli31* | | (2) | DP-6147 |
| Δ*rli31* + pIMK:*rli31* | | (2) | DP-6351 |
| Δ*rli31* + pIMK:*rli31* Mutant A | | This Study | DP-6352 |
| Δ*rli31* + pIMK:*rli31* Mutant B | | This Study | DP-6353 |
| Δ*rli31* + pIMK:*rli31* Mutant A+B | | This Study | DP-6354 |
| Δ*rli31* + pIMK:*rli31* Mutant C | | This Study | DP-6355 |
| Δ*rli31* + pIMK:*rli31* Mutant D | | This Study | DP-6356 |
| Δ*rli31* + pIMK:*rli31* Mutant C+D | | This Study | DP-6357 |
| Δ*rli31* + pIMK:*rli31* Mutant E | | This Study | DP-6358 |
| Δ*pgdA* | | (2) | DP-5188 |
| Δ*pgdA* lysozyme resistant suppressor #1 | | This study | DP-6359 |
| Δ*pgdA* lysozyme resistant suppressor #2 | | This study | DP-6360 |
| Δ*pgdA* lysozyme resistant suppressor #3 | | This study | DP-6361 |
| Δ*pgdA* lysozyme resistant suppressor #4 | | This study | DP-6362 |
| Δ*pgdA* lysozyme resistant suppressor #5 | | This study | DP-6363 |
| Δ*spoVG* | | This study | DP-6364 |
| Δ*spoVG-ORF* | | (3) | DP-6365 |
| Δ*rli31* Δ*spoVG* | | This study | DP-6366 |
| Δ*spoVG* Δ*pgdA* | | This study | DP-6367 |
| *rli31::TN917* | | (2) | DP-6151 |
| Δ*spoVG* swarming suppressor #1 | | This study | DP-6368 |
| Δ*spoVG* swarming suppressor #2 | | This study | DP-6369 |
| Δ*spoVG* swarming suppressor #3 | | This study | DP-6370 |
| Δ*spoVG* swarming suppressor #4 | | This study | DP-6371 |
| Δ*spoVG* swarming suppressor #5 | | This study | DP-6372 |
| Δ*spoVG* swarming suppressor #6 | | This study | DP-6373 |
| *E.coli BL21* with pET20b – *spoVG:*6His | | This study | DP-6374 |

1. **Becavin C, Bouchier C, Lechat P, Archambaud C, Creno S, Gouin E, Wu Z, Kuhbacher A, Brisse S, Pucciarelli MG, Garcia-del Portillo F, Hain T, Portnoy DA, Chakraborty T, Lecuit M, Pizarro-Cerda J, Moszer I, Bierne H, Cossart P.** 2014. Comparison of widely used Listeria monocytogenes strains EGD, 10403S, and EGD-e highlights genomic variations underlying differences in pathogenicity. MBio **5:**e00969-00914.

2. **Burke TP, Loukitcheva A, Zemansky J, Wheeler R, Boneca IG, Portnoy DA.** 2014. Listeria monocytogenes is resistant to lysozyme through the regulation, not the acquisition, of cell wall-modifying enzymes. J Bacteriol **196:**3756-3767.

3. **Whiteley AT, Pollock AJ, Portnoy DA.** 2015. The PAMP c-di-AMP Is Essential for Listeria monocytogenes Growth in Rich but Not Minimal Media due to a Toxic Increase in (p)ppGpp. Cell Host Microbe.
