# Supplementary material for: SpoVG Is a Conserved RNA-Binding Protein That Regulates Listeria monocytogenes Lysozyme Resistance, Virulence, and Swarming Motility
Source: mBio. 2016 Apr 5;7(2):e00240-16. doi: 10.1128/mBio.00240-16 (PMC4959528; doi:10.1128/mBio.00240-16)
Supplement: Table S3 — Oligonucleotides used in this study. [file mbo002162736st3.docx]

| **Supplemental Table 3: Oligonucleotides used in this study** | |
| --- | --- |
| **Name** | **Sequence** |
| TB140: rli31 promoter Forward with EagI | attaCGGCCG gccaattcctcctatatataagat |
| TB141: rli31 Reverse with SalI | attaGTCGAC cctcattttcagagcatctcta |
| TB14: Rli31 Mutant A Forward | cccatagagattgtcaggggaaataagctaattgaaaataaa |
| TB15: Rli31 mutant A Reverse | tttattttcaattagcttatttcccctgacaatctctatggg |
| TB16: Rli31 mutant B Forward | cttaattgcttatttcccctaaacactcctttaagatag |
| TB17: Rli31 mutant B Reverse | ctatcttaaaggagtgtttaggggaaataagcaattaag |
| TB18: Mutant D Forward | gatagttgtagcacCCaatttcccagattGGg |
| TB19: Mutant D Reverse | cCCaatctgggaaattGGgtgctacaactatc |
| TB20: Mutant C Forward | cagaatttcccagattgggtgctataattatgtagaaatag |
| TB21: Mutant C Reverse | ctatttctacataattatagcacccaatctgggaaattctg |
| TB22: Mutant E Forward | gaaaataaattactttttccatagaaatattcttaattgcttatttc |
| TB23: Mutant E Reverse | gaaataagcaattaagaatatttctatggaaaaagtaatttattttc |
| TB211: lmo0196/7 deletion A - F- BamHI | tca ggatcc gaataactgcaggaaccattatattctcct |
| TB212: lmo0196/7 deletion B - Rev | tgaaaattttaaattattcagcagaaacggtattcacgtaaaattcttccctatgaaca |
| TB213: lmo0196/7 deletion C -Forw | tgttcatagggaagaattttacgtgaataccgtttctgctgaataatttaaaattttca |
| TB214: lmo0196/7 deletion D - Rev – SalI | gta gtcgac ctacaatgcctaggtcatcacgagataac |
| TB216: lmo0196 Forward, for qPCR | gtgagattacgacgtgttgagaca |
| TB217: lmo0196 Reverse, for qPCR | ggatgagcgatatctctaaactcac |
| TB254: SpoVG I F with Nde1, for pET20b | GCGCCATATGatggagattacagatgtgagattacgac |
| TB255: SpoVG I R, w Xho1, for pET20b | tatactcgaggttttcttctacaatactttcgtctgc |
| TB377: cap41 probe Forw | GAGTATAATTATTTTTAATTTACATATAAATAAAAAGGCGAAAATAATGCGGTTTAAAAGTAATTAAT |
| TB378: cap41 probe Rev | ATTAATTACTTTTAAACCGCATTATTTTCGCCTTTTTATTTATATGTAAATTAAAAATAATTATACTC |
| TB379: pgdA promoter 1 | acaaaaactagaaacttgccttttttcatgtataattgttttataagaa |
| TB381: pgdA promoter 2 | tttgttaagaaatcgttggtccttttttcctattttagtacaaaaacta |
| TB383: pgdA promoter 3 | cggtaaaagctcccactttatccgtgtcttttttgtgacatttgttaag |
| TB389: pgdA ORF | gtcgcgcaacaaagtaataatgcagatggacagactaatgaaagaccag |
| TB391: scrambled probe 1 | AATTCGATATATGATTTTGAAAATGAATTTCATAACTTTTAAATTGCGT |
| TB393: scrambled probe 2 | GCCGAGCGGGTACGCCGTGCGGTATGGGCCCACGCTCCGTGGCGTGCCG |
| TB395: Scrambled probe 3 | ATTTTTTAGCGCTATAAAAAAGCTATTTTTTAAAAAAGTACTTTTTTGA |
| TB401: rli32 F with T7 promoter for IVT | Ccaagtaatacgactcactatagggtggagagctttcatttttccc |
| TB402: rli32 R, for IVT | caaaaaaataaccgcaccagggg |
| TB403: 6S RNA, with T7, for IVT | Ccaagtaatacgactcactatagggaaaagaaaccctaatgtattcg |
| TB404: 6S RNA, Reverse, for IVT | caaaaaagaaaccccaatcgtaccg |
| TB149: rli31 F with T7 promoter for IVT | Ccaagtaatacgactcactata gg tatcccatagagattgtcatttgaaataag |
| TB150: rli31 R, for IVT | taattatagcacagaatctgggaaattctg |
| TB413: Rli31 F, T7 for mut A, A+B IVT | Ccaagtaatacgactcactata gg tatcccatagagattgtcaGGG |
| TB455: SRP, Forw, with T7 for IVT | Ccaagtaatacgactcactataggttgtcgtgctagacggggaggta |
| TB456: SRP Rev, for IVT | ttagtgtcgcgcacctcacatcga |
| TB457: Rli109, Forw, with T7 for IVT | Ccaagtaatacgactcactatagggcagtggaattaaaggcatctaag |
| TB458: Rli109, Rev for IVT | tgtcactcgcaaaagcattgc |
| TB447: RliI, with T7, Forward, for IVT | Ccaagtaatacgactcactataggtgagatgacatgtttcttttgaatg |
| TB448: RliI, reverse, for IVT | ttttttcagacaacaaaaaagcg |
